# Nutrient variation shapes survival without altering activity or social interaction networks in a sawfly

**DOI:** 10.1093/beheco/arag102

**Published:** 2026-08-26

**Authors:** Steven Janz, Caroline Müller, Pragya Singh

**Affiliations:** Chemical Ecology, Bielefeld University, Universitätsstr. 25, Bielefeld 33615, Germany; Chemical Ecology, Bielefeld University, Universitätsstr. 25, Bielefeld 33615, Germany; Joint Institute for Individualisation in a Changing Environment (JICE), University of Münster and Bielefeld University, PO Box 100131, Bielefeld 33501, Germany; Chemical Ecology, Bielefeld University, Universitätsstr. 25, Bielefeld 33615, Germany

**Keywords:** macronutrient, adult nutrition, behavioral repeatability, social interactions, holometabolous insect, herbivore

## Abstract

Nutrient availability can shape key life-history traits including behavior. However, many studies of nutrient-dependent behavior have focused on individual activity, whereas the effects of specific nutrients on social interactions and the resulting networks remain poorly understood, especially in insects that are neither eusocial nor solitary. As major nutritional components, amino acids and carbohydrates can influence individual condition, such that variation in the nutrient availability may alter individual activity, social interactions, and ultimately lifespan. We examined how variation in either amino acids or carbohydrates influenced activity and its short-term repeatability, social interaction networks (based on mating and agonistic interactions), and lifespan in adults of the turnip sawfly, *Athalia rosae*, which naturally encounter heterogeneity in these nutrients across the nectar of their host plants. We provided adults with different diets spanning ecologically realistic concentrations of either amino acids or carbohydrates, and recorded adult activity over 2 d along with social interactions in mixed-sex groups. We found that neither activity nor its short-term repeatability, ie the consistency of activity across the assay days, differed across individuals from different dietary treatments. Social interactions and the resulting networks also did not differ significantly among individuals from different diets, but high-carbohydrate diets extended lifespan, whereas amino acid variation had no detectable effect on survival. These findings suggest that short-term differences in nutrient availability can influence lifespan but not individual activity or social interactions.

## Teaser

Diet can shape how animals behave and how long they live, but its effects on social interactions are less well understood. We fed sawflies nectar-like diets that differed in amino acids or carbohydrates. Diet did not affect activity or its short-term repeatability, or social interaction networks based on mating and agonistic interactions. Instead, nutrients influenced sawfly lifespan, with carbohydrate-rich diets increasing lifespan. This suggests that nutrition can affect survival without altering activity or social interactions.

## Introduction

Animals vary widely in how often and intensely they interact with others. This heterogeneity can influence processes such as information flow, mating success and disease transmission, and may even shape life-history traits of individuals (Frank 2007; Krause et al. 2015; Fisher et al. 2019). Individuals that interact frequently may face larger energetic or social costs, while those with fewer interactions may have limited access to resources or mates (Archer 1988; Briffa and Sneddon 2007; Snyder-Mackler et al. 2020). Identifying the factors that shape variation in interaction frequency is important for understanding how social structure emerges and what consequences such variation may have. One such factor is an individual’s nutrient intake, which can affect its nutritional and energetic condition and thereby influence behaviors such as foraging, movement and mating, for example by altering activity levels, mate-searching efforts and resource acquisition (Raubenheimer and Simpson 2018; Gruhn et al. 2021; Raubenheimer et al. 2023). Furthermore, nutritional differences may contribute to modulating among- and within-individual variation in activity if the energetic state influences behavioral performance across repeated observations (Tremmel and Müller 2013; Royauté and Dochtermann 2017; Moran et al. 2021). For example, in the southern field cricket, *Gryllus bimaculatus*, juvenile diet altered both mating activity and behavioral stability, with males raised on a high-protein diet being more active during mating but less behaviorally stable than males raised on a carbohydrate-rich diet (Han and Dingemanse 2017). Moreover, as interactions themselves require energy and motivation, diet-linked shifts in condition may also alter how individuals encounter and interact with conspecifics, potentially influencing the structure of social interaction networks or even ecological networks (Senior et al. 2016; Lihoreau et al. 2018; Cuff et al. 2024). Thus, examining diet effects on individuals at the social interaction network level can reveal structured variation in interaction patterns that is not detectable from pairwise interactions alone, and can link individual behavioral differences to their broader ecological consequences (Krause et al. 2010).

Studies on social insects highlight how nutrition can alter interaction patterns. For example, in the black garden ant, *Lasius niger*, variation in protein and carbohydrate intake modifies recruitment behavior and foraging patterns (Portha et al. 2002). Among the carpenter ants, *Camponotus fragilis*, workers under food limitation interact with more individuals and travel larger distances than those without food limitation, indicating that nutritional context can shift the structure of within-group social contacts (Guo et al. 2024). Although these studies demonstrate that diet can influence how individuals interact, experimental tests linking diet composition to social interaction network structure remain scarce.

Such gaps are especially apparent in animals that associate with conspecifics but lack stable groups, relationships, or affiliative interactions. Such species can form temporary aggregations on shared sites, such as for feeding or reproduction, creating opportunities for repeated interactions even without stable social groups (Cocroft 2005; Wäckers et al. 2007; Stanton et al. 2016; Tóth et al. 2020). If these feeding sites vary nutritionally, diet-mediated differences in condition or motivation may influence individual activity, in turn impacting how often individuals encounter one another as well as the social interaction networks arising from these interactions (Wäckers et al. 2007). Shifts in interaction frequency can also affect other traits, including lifespan. For example, frequent mating or aggressive interactions may impose energetic costs (Singh et al. 2024), whereas fewer interactions may reduce access to resources (Snyder-Mackler et al. 2020). Thus, nutrition may influence survival both directly and indirectly through its effects on social behavior.

More broadly, nutrient availability in food sources is often multidimensional, varying not only in total nutrient concentration but also in the diversity and composition within a nutrient class, such as the identity and relative proportions of individual amino acids or sugars (Raubenheimer and Simpson 2018; Nicolson 2022). Such nutritional variation can be studied within the nutritional geometric framework, a multidimensional approach for examining how individuals balance the intake of multiple nutrients to achieve optimal survival, growth, and/or reproduction (Raubenheimer and Simpson 2018). Most empirical tests of this framework focus on the consequences of nutrient ratios, usually proteins relative to carbohydrates (Anderson et al. 2020). However, animals may also experience variation in the availability (or concentrations) of either proteins or carbohydrates rather than coordinated shifts in both nutrient classes (Wäckers et al. 2007; Wilson et al. 2019). For example, adult herbivorous insects frequently rely on nectar as a primary energy source, and this resource can vary markedly in amino acid or/and carbohydrate concentration and composition, but also in lipids, minerals and other metabolites present in low concentrations, both among and within plant species (Nicolson and Thornburg 2007; Nicolson 2022). Such variation may affect not only survival but also the capacity for sustained activity, interactions, and reproduction. Examining how individuals respond behaviorally to variation in nutrient availability is therefore crucial for linking plant-level resource heterogeneity to animal behavioral ecology.

We addressed this gap using the turnip sawfly *Athalia rosae*, an insect herbivore whose adults feed on nectar of Apiaceae plants that varies widely in amino acid and carbohydrate concentrations (Venjakob et al. 2022). The adults of *A. rosae* congregate both on Apiaceae plants for nectar and on Lamiaceae plants (eg *Ajuga reptans*) for acquisition of specific metabolites (neo-clerodane diterpenoids or clerodanoids) for nonnutritional purposes, such as increased mating success and defense against predation (Nishida et al. 1989; Brueggemann et al. 2023; Singh et al. 2024; Singh and Müller 2025). Such sites can then create opportunities for repeated encounters, including mating and agonistic contacts, from which short-term social interaction networks can be quantified. By offering low or high concentrations of either amino acids or carbohydrates separately in compositions representative of the natural range in nectar of Apiaceae, and holding nutrient composition constant within each dietary treatment, we tested whether variation in nutrient availability and concentration influences (i) individual behavioral activity and its repeatability, (ii) the structure of social interaction networks, and (iii) adult lifespan. We predicted that individuals provided with a high-carbohydrate diet would exhibit the highest activity, as carbohydrates are expected to provide readily available energy for movement and interactions (Bretscher and O’Connor 2020). Moreover, we predicted that these individuals would have more social interactions, potentially due to their higher activity. Finally, as adult *A. rosae* rely on nectar-derived resources and carbohydrates can provide readily available energy for adult maintenance, we expected the high-carbohydrate diet to extend lifespan relative to the other treatments.

## Materials and methods

### Study organism

*A. rosae* individuals used in this experiment were taken from a laboratory population that was established using adults collected in and around the surrounding of Bielefeld, Germany, and supplemented annually with wild-caught adults. The laboratory stock was maintained in mesh cages (60 × 60 × 60 cm) under a 16:8 h light:dark cycle, at room temperature (15 to 25 °C) and approximately 60% relative humidity (see Singh et al. 2022 for details). Several males (∼30) and females (∼40) were released in each cage and provided with *Sinapis alba* (Brassicaceae) plants for oviposition. Emerging larvae were reared on *Brassica rapa* var. *pekinensis* (Brassicaceae). Plants of *S. alba* and *B. rapa* were cultivated from seeds in a climate chamber (20 °C, 16:8 h light:dark, 70% relative humidity) and a greenhouse (≥20 °C, 16:8 h light:dark, 70% relative humidity), respectively. Adult *A. rosae* are relatively short-lived, typically surviving for 1 to 3 wk at ∼20 °C, although life-history traits may vary with individual nutritional and social conditions (Paul et al. 2019; Singh et al. 2024; Brueggemann et al. 2025).

Over multiple days, freshly eclosed adults were collected, sexed, and randomly assigned to 1 of 5 dietary treatments: (i) control (distilled water only); (ii) low amino acid (2 mg mL^−1^, mixture of 16 amino acids); (iii) high amino acid (15 mg mL^−1^); (iv) low carbohydrate (5 mg mL^−1^, mixture of fructose, glucose and sucrose); and (v) high carbohydrate (150 mg mL^−1^) (Fig. 1i). Each eclosed adult was weighed (female: 21.7 ± 5.9 mg, male: 11.9 ± 3.6 mg, Figure S1) on a calibrated analytical balance (Sartorius CPA224S-0CE, Sartorius AG, Göttingen, Germany) and placed individually in a Petri dish (55 mm diameter) with filter paper and a tissue with dietary solution. Each day, 200 µL of the assigned dietary solution was added to the tissue, and the tissues were replaced every 2 to 3 d. The concentrations of amino acids and carbohydrates in the different dietary treatments were chosen based on the highest and lowest species-specific averages reported by Venjakob et al. (2022), each representing the mean of multiple individuals of different Apiaceae plants, to simulate the natural range of conditions. The amino-acid diets consisted of a mixture of 16 amino acids, whereas the carbohydrate diets consisted of a mixture of fructose, glucose, and sucrose. The respective nutrient composition was kept constant within each dietary treatment. For detailed dietary treatment composition, see Tables S1 and S2.

**Figure 1:**
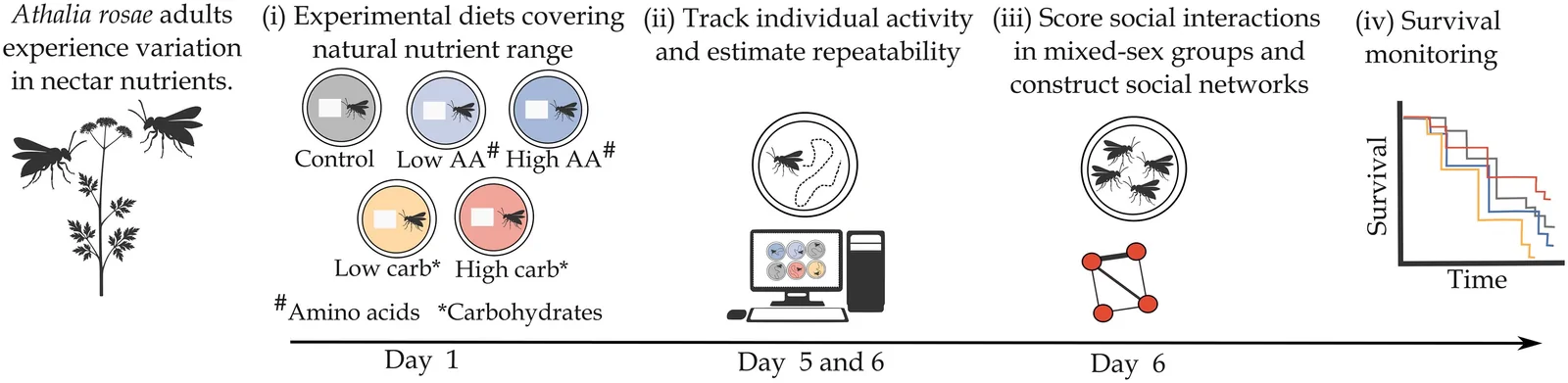
Overview of the experimental design using *Athalia rosae* adults. Schematic illustration of the study showing i) dietary treatments spanning natural ranges of amino acids and carbohydrates (individuals were kept on their respective diet throughout), ii) individual activity assays on days 5 and 6 to quantify movement and estimate behavioral repeatability, iii) social interaction (based on mating and agonistic interactions) assays in mixed-sex groups used to construct social interaction networks, and iv) subsequent monitoring of adult survival.

### Activity assay and repeatability estimates

Five days after the start of the dietary treatment, the activity level of each individual was recorded. For the activity assay, each individual was transferred to a clean Petri dish (55 mm diameter) and recorded for 60 min using a digital camera mounted above the setup, with 6 individuals filmed simultaneously per trial (Fig. 1ii). Activity levels were quantified using automated video tracking software (AnimalTA, 2024, Version 3.2.2; Chiara and Kim 2023) from which the proportion of time spent moving was extracted. We set a movement-speed threshold of 0.8 cm/s, only above which the individual was considered to move to avoid counting minor displacements, eg caused by tracking imprecision or posture-related movements. The threshold was chosen after visually inspecting the videos. After the activity assay, individuals were returned to the climate cabinet. On the 6th day of dietary treatment, the activity assay was repeated to allow us to estimate short-term repeatability of activity (proportion of time spent moving) across 2 consecutive days. See Table 1 for sample size for the activity assay and repeatability estimates.

**Table 1:** Short-term repeatability of activity across day 5 and day 6 in adult female and male *Athalia rosae* across dietary treatments.

Sex	Treatment	*N* (*n*)	Posterior mean (R)	95% CrI
Female	Control	18 (17)	0.43	0.21 to 0.67
	Low amino acid	15 (15)	0.58	0.31 to 0.79
	High amino acid	15 (14)	0.42	0.21 to 0.64
	Low carbohydrate	16 (15)	0.38	0.15 to 0.66
	High carbohydrate	16 (16)	0.43	0.23 to 0.64
Male	Control	13 (13)	0.50	0.26 to 0.73
	Low amino acid	13 (12)	0.53	0.25 to 0.77
	High amino acid	15 (14)	0.52	0.25 to 0.76
	Low carbohydrate	14 (13)	0.39	0.19 to 0.62
	High carbohydrate	16 (16)	0.51	0.28 to 0.73

Repeatability (*R*) was estimated separately for females and males. Values shown are posterior means with 95% credible intervals (CrI). *N* (*n*) indicates the activity-assay sample size and, in parentheses, the repeatability-analysis sample size. The repeatability sample size was smaller in some treatments because some individuals died before the second assay.

### Group- and individual-level social interaction network measures

After the activity assay, the individuals were used for a social interaction network assay. For each replicate, 2 males and 2 females from the same dietary treatment and adult eclosion date were color-marked 1 h before the behavioral assay (Vdstar, China) on their thorax for individual identification during behavior scoring. The colors were alternated between sexes and treatments across replicates to avoid any color effects. This method has been used previously for social interaction network analysis of *A. rosae* (Singh et al. 2024). To start the assay, the 4 color-marked individuals (2 males and 2 females) were placed together in a clean Petri dish (Fig. 1iii) and recorded for a duration of 30 min using a digital camera mounted above the setup with up to 6 groups filmed simultaneously per trial. Social interactions were quantified using the behavioral observation software BORIS (Friard and Gamba 2016), while being blind to the dietary treatment. Mating and agonistic interactions were scored for each individual in a group replicate. Mating interactions were scored when copulation occurred, with copulation defined as starting when the genitalia became attached (male inserts genitalia into the female; Paul and Müller 2022). Agonistic interactions comprised behaviors involving physical contact, including foreleg battling, fighting with the use of all limbs and full-body movements, as well as “nibbling”, in which the attacker's mouthparts contacted the defender's body (Paul and Müller 2022). Some individuals died before the social interaction network assay (see Table S3), resulting in some filmed groups containing fewer than 4 individuals. Groups in which 1 or more individuals had died before the assay (0 to 5 per dietary treatment group) were excluded from the social interaction network analyses. However, these individuals were retained in the lifespan analyses (see below). We repeated the above setup to get more replicates for all treatments so as to obtain 15 complete 4-individual group replicates for each dietary treatment in the social interaction network assay. The activity and social interaction network assay were conducted in the laboratory at room temperature (20 to 25 °C), approximately 60% relative humidity, under uniform lighting and usually between 11 AM and 4 PM. After the social interaction network assay, individuals were returned to the climate chamber.

### Survival across dietary treatments

For the experimental individuals used for the initial set-up (see above), survivorship was checked daily. We did not monitor the lifespan of individuals used in the additional replicates established to obtain 15 complete social interaction network replicates per treatment, resulting in a sample size of 18 individuals per sex for the control treatment and 16 individuals per sex for all other treatments for the survival assay (see Table S3). All individuals were housed in a climate cabinet (20 °C, 16:8 h light:dark, 60 to 65% relative humidity) except during the experimental assays and had access to their assigned diet.

### Statistical analyses

All analyses were done in R version 4.3.3 (R Core Team 2024). We analysed the proportion of time individuals spent moving using a beta regression mixed model (logit link function), with body mass, treatment, sex, the interaction between treatment and sex, and day as fixed effects, with individual identity and video identity included as random effects (glmmTMB package version 1.1.11, Brooks et al. 2017).

To estimate short-term repeatability of activity (proportion of time spent moving) across the 2 assay days and to compare repeatability across dietary treatments, we analysed females and males separately using Bayesian multivariate mixed models in MCMCglmm (Hadfield 2010). Only individuals with activity measurements on both day 5 and day 6 were included. As the response variable was the proportion of time spent moving, values were logit-transformed after constraining any exact 0 or 1 value slightly inward. Within each sex, we fitted a 5-response Gaussian model, with 1 response corresponding to each dietary treatment. The fixed-effects structure included treatment-specific intercepts and treatment-specific day effects, and individual identity was fitted as a treatment-specific random intercept. Residual variance was also estimated separately for each treatment. We used weakly informative inverse-Wishart priors for both the random and residual variance structures (*V* = diag(5), nu = 5.002) and ran models for 260,000 iterations with a burn-in of 60,000 and thinning interval of 200. Convergence was assessed from trace plots, autocorrelation and effective sample sizes. For each posterior sample, treatment-specific repeatability was calculated as *R* = *V*_ID_/(*V*_ID_ + *V*_R_), where *V*_ID_ is the among-individual variance and *V*_R_ the residual variance. We then compared dietary treatments within each sex by calculating posterior differences in repeatability (Δ*R*) for all pairwise treatment contrasts. We report posterior means and 95% credible intervals.

For the social interaction network assay, we quantified both group- and individual-level properties of the network. For each replicate, we first assembled an undirected, weighted social interaction network in which edges represented the total number of interactions between each dyad, pooling mating and agonistic contacts to describe overall interaction intensity. Both mating and agonistic interactions were treated as nondirectional, ie we did not distinguish between which individual initiated the interaction, for constructing the networks. The social interaction networks analysed here represent short-term interaction structure derived from mating and agonistic contacts in standardized mixed-sex groups, rather than affiliative relationships or stable long-term social associations. We additionally constructed separate weighted networks for mating interactions only and agonistic interactions only, because these behaviors may differ in their response to dietary treatment. For each replicate network, at the group level, we quantified the number of interactions and the number of interacting dyads. For the social (pooled) and agonistic networks, dyads were considered out of 6 possible dyads per group; for mating networks, dyads were considered out of 4 possible mixed-sex dyads. Next, using these networks, we computed a node-level (individual-level) metric, namely strength, for every individual within each replicate. The individual-level network strength is calculated as the sum of the weights of all edges connected to an individual, which provides a measure of how actively an individual engaged with others. All network calculations were carried out in igraph (‘igraph’ package version 2.2.1, Csárdi et al. 2023). We did not apply a network randomization or null-model procedure as our aim was to test whether experimentally assigned dietary treatment affected observed network summary measures across replicate groups, rather than whether each network departed from a randomized structural expectation. As all replicate networks were generated under a standardized design, we analysed the observed group- and individual-level metrics directly.

We analysed the dietary treatment effect on the number of interactions separately for social, mating-only, and agonistic-only interaction networks, using a negative binomial generalized linear model (log link) with treatment as the fixed effect (MASS package version 7.3-60.0.1; Venables and Ripley 2002). To examine the effect of dietary treatment on the number of interacting dyads for social, mating-only, and agonistic-only networks, we fitted a zero-inflated binomial generalized linear model with the number of interacting dyads out of the relevant number of possible dyads as the response and treatment as a fixed effect (glmmTMB package version 1.1.11, Brooks et al. 2017). The effect of dietary treatment, sex and their interaction on individual-level network strength for social and agonistic-only networks was analysed using a negative binomial mixed model (log link) with group identity as a random effect. We did not analyse individual-level network strength for mating-only networks, because although mating strength can differ among individuals within a replicate, every mating interaction involves 1 male and 1 female. Therefore, within each replicate group, the total mating strength of males and females is necessarily the same, and because each group contained 2 males and 2 females, the mean mating strength of males and females is also the same. Thus, sex differences and treatment-by-sex differences in mating strength cannot be tested meaningfully for mating-only networks, while treatment-level differences are already captured by the group-level number of mating interactions.

To examine the effect of dietary treatment on adult lifespan, survival time was analysed with sex-specific Cox proportional hazards models (survival package version 3.8-3, Therneau 2024; survminer package 0.5.1, Kassambara et al. 2021) including dietary treatment as a fixed factor, followed by Tukey-adjusted post-hoc comparisons among treatments (emmeans package 2.0.0, Lenth 2023).

## Results

### No effect of diet on activity in both sexes

For the proportion of time spent moving (Fig. 2), there was no significant effect of dietary treatment (*χ*^2^ = 5.59, df = 4, *P* = 0.232), sex (*χ*^2^ = 0.03, df = 1, *P* = 0.872), their interaction (*χ*^2^ = 5.20, df = 4, *P* = 0.267) or of body mass (*χ*^2^ = 2.87, df = 1, *P* = 0.090). There was a significant effect of day with individuals being more active on day 5 than day 6 (*χ*^2^ = 4.42, df = 1, *P* = 0.035).

**Figure 2:**
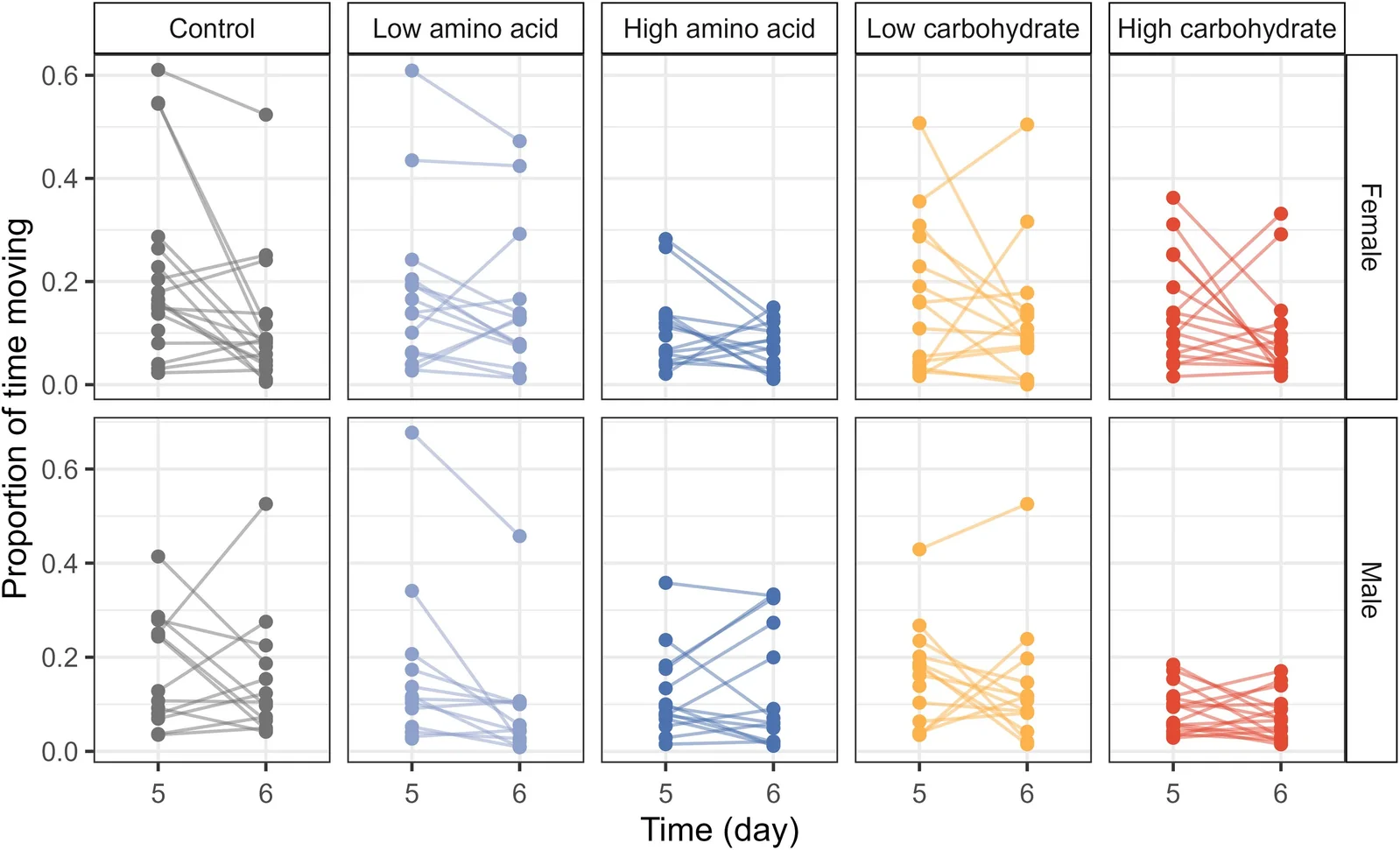
Proportion of time spent moving for females (top row) and males (bottom row) during activity assays across the 5 dietary treatments. Points show raw data for each adult on days 5 and 6, with lines connecting observations from the same individual.

### Activity repeatability did not differ among individuals of the different dietary treatments

Activity showed moderate short-term repeatability across the 2 assay days in both sexes (Table 1). In females, posterior mean repeatability estimates ranged from 0.38 to 0.58 across dietary treatments, whereas in males they ranged from 0.39 to 0.53. However, pairwise posterior comparisons of repeatability among individuals of different dietary treatments (ΔR) provided little evidence for dietary impacts on either sex, as all 95% credible intervals for ΔR overlapped with zero (Table S4). Thus, activity was repeatable over the 2 assay days, but repeatability itself was not altered by dietary treatment.

### Diet did not alter social interaction networks

At the group level, there was no significant effect of dietary treatment on the number of social interactions (*χ*^2^ = 3.75, df = 4, *P* = 0.44; Fig. 3a) or on the number of interacting dyads (*χ*^2^ = 7.92, df = 4, *P* = 0.09; Fig. 3b, see also Figure S2) for the social interaction networks. At the individual-level, there was also no significant effect of dietary treatment (*χ*^2^ = 2.75, df = 4, *P* = 0.60), sex (*χ*^2^ = 1.23, df = 1, *P* = 0.27), or their interaction (*χ*^2^ = 1.84, df = 4, *P* = 0.77) on the strength of social interactions (Fig. 3c, d). Similarly, there was no significant effect of dietary treatment on the number of interactions or number of interacting dyads for both mating-only and agonistic-only interaction networks. Neither was there a significant effect of dietary treatment, sex, or their interaction on the strength of interactions for agonistic-only interaction networks (all *P* > 0.05; see Table S5, Figures S3 and S4).

**Figure 3:**
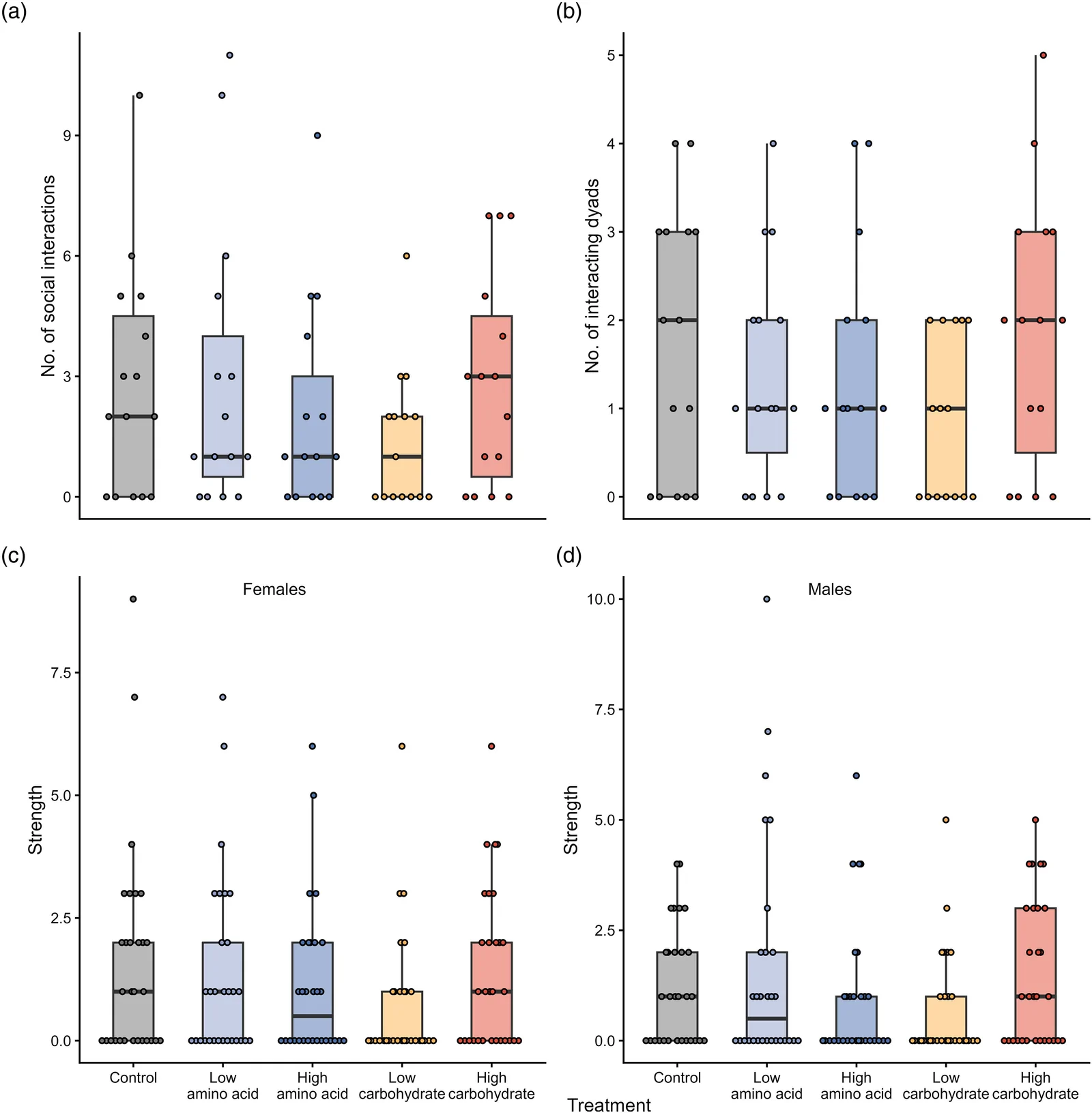
Effect of dietary treatment on social interaction (based on mating and agonistic interactions) networks in adult *Athalia rosae*. a) Number of social interactions and b) number of interacting dyads per group. Individual strength for c) females and d) males, respectively. Boxplots show the median, 25th and 75th percentiles, with whiskers indicating values within 1.5 times the interquartile range. Colored circles denote individual data points.

### High-carbohydrate diet increased lifespan

Dietary treatment influenced adult lifespan in both females (likelihood ratio: *χ*^2^ = 29.68, df = 4, *P* < 0.001; Fig. 4a) and males (*χ*^2^ = 33.19, df = 4, *P* < 0.001; Fig. 4b). In both sexes, sawflies on the high-carbohydrate diet showed a significantly longer lifespan than those of other treatments (all *P* ≤ 0.001), while no significant differences were detected among individuals of these other dietary treatments (all *P* > 0.99) (Tables S6 and S7).

**Figure 4:**
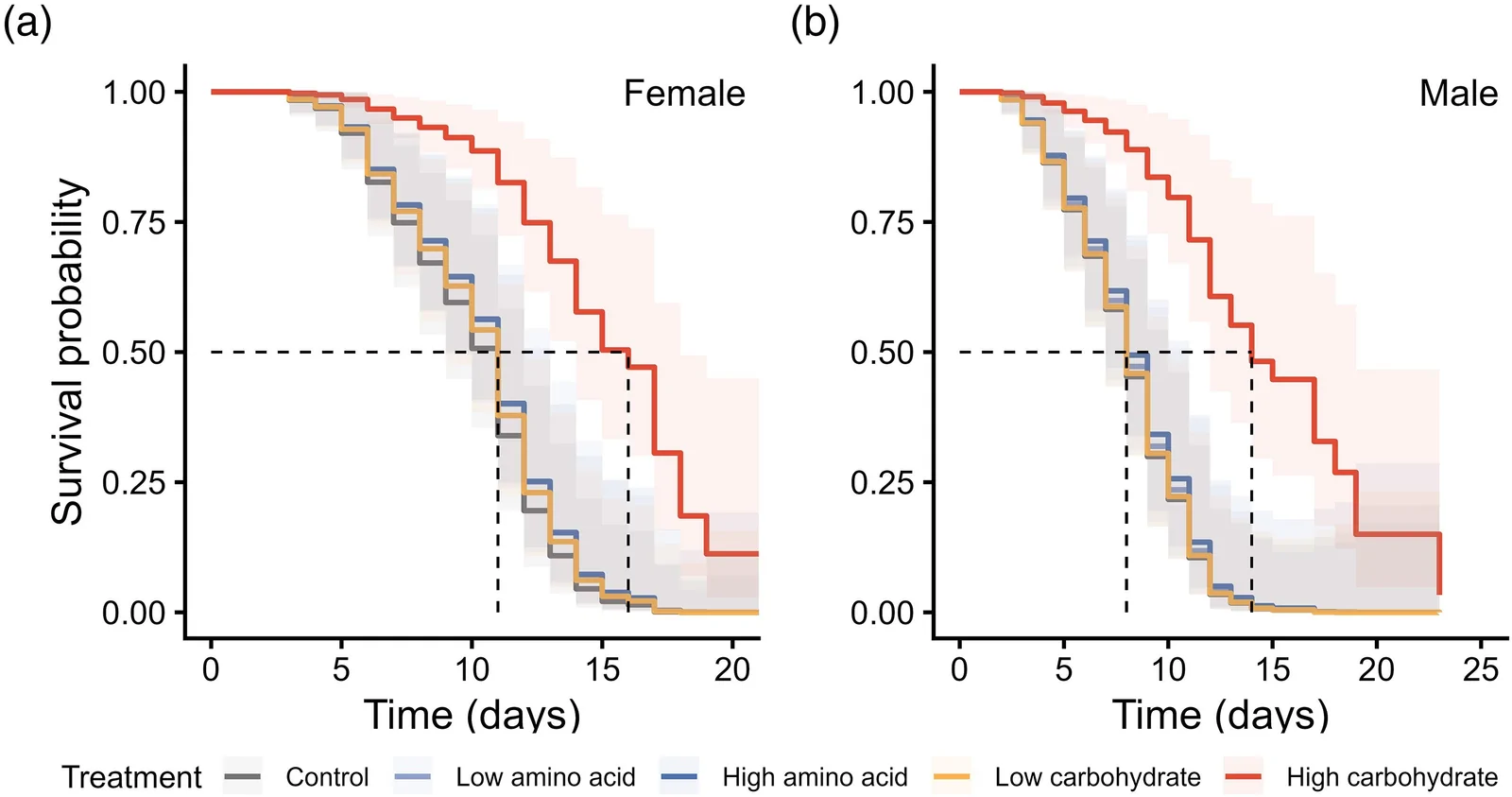
Cox model-predicted survival curves showing dietary treatment effects on adult lifespan in a) female and b) male *Athalia rosae*. Shaded regions represent 95% confidence intervals, and dashed lines indicate the 50% survival level and median survival time.

## Discussion

By manipulating amino acid and carbohydrate availability and concentration across naturally realistic ranges, our study examined how variation in single nutrient classes shapes behavioral expression, interaction patterns and survival in an herbivorous insect. A key result was that nutrient availability did not alter overall activity levels. Across treatments, individuals maintained similar proportions of time spent moving, suggesting that *A. rosae* may sustain baseline activity levels even when energetic input is constrained. Alternatively, the absolute ranges of amino acids and carbohydrate tested, although ecologically relevant, may fall within a tolerance window that supports similar behavioral expression during the studied period, even if energetic reserves ultimately diverge. The significant lifespan differences among treatments support the latter possibility. Similar patterns have been observed in other insects, where activity remains stable despite nutritional variation, such as in the hover fly *Eristalis tenax,* which maintains consistent locomotor levels across dietary treatments (Thyselius and Nordström 2016), and in the Colorado potato beetle, *Leptinotarsa decemlineata*, where walking behavior shifts only after extreme, prolonged deprivation while remaining unchanged across ecologically relevant starvation periods (Gui and Boiteau 2010). Interestingly, in *E. tenax* there was also a strong diet-dependent effect on survival despite no detectable change in activity. With regard to *A. rosae*, adults may diversify their nectar uptake among plant individuals and species under natural conditions to maintain an optimal nutrient intake, as shown, for example, in bumblebees (Selva et al. 2025).

Our study design isolated concentration effects within single nutrient classes and therefore addresses only one dimension of nutrient availability. In natural nectar, however, amino-acid and sugar profiles as well as the composition of other constituents, such as lipids, minerals, vitamins, proteins, and specialized metabolites, may vary both among and within species (Nicolson and Thornburg 2007; Nicolson 2022; Venjakob et al. 2022). Such multidimensional variation, in concentration and diversity of composition, could generate different behavioral or survival outcomes than those observed here (Raubenheimer and Simpson 2018; Anderson et al. 2020).

Moreover, while diet did not alter mean activity levels of *A. rosae*, activity was lower on day 6 than on day 5. In invertebrates, repeated behavioral assays can modify responses through habituation and novelty effects, and activity and exploratory behavior in arenas may decline with repeated exposure (Soibam et al. 2013; Müller and Müller 2017). Alternatively or in addition, there can also be age-related change with reduced activity of older individuals (Promislow et al. 2022). In our setup, we cannot distinguish between the 2 potential explanations. Although overall activity was reduced on the second testing day, activity was repeatable across the 2 consecutive assay days in both sexes, indicating short-term behavioral consistency. There was little evidence that amino acid or carbohydrate availability altered repeatability itself, as all pairwise ΔR credible intervals overlapped zero. However, low-amino-acid females had the highest posterior mean repeatability, with weak-to-moderate directional support for higher repeatability than the females of the other noncontrol treatments, which may suggest a potential treatment-specific pattern that should be explored in future studies. Studies in other insects show that diet can shape behavioral stability, but the magnitude and direction of such effects vary across species and nutrient axes (Han and Dingemanse 2017; Royauté and Dochtermann 2017).

In contrast to expectations based on studies in social insects, in which nutritional changes can restructure interactions (Portha et al. 2002; Guo et al. 2024), we found no evidence that diet influenced either group-level or node-level metrics in the short-term social (sum of mating and agonistic interactions), mating-only or agonistic-only interaction networks measured in our setup. The absence of treatment effects on the number of interacting dyads, interaction frequencies, or strength indicates that the interactions of *A. rosae* are resilient to variation in single nutritional classes during our observation period. Moreover, if social interactions arise from differences in activity levels, then this would be consistent with the observed lack of differences in activity patterns. This contrasts with the strong effect of nonnutritional plant-derived chemicals, such as clerodanoids, in *A. rosae*, which can markedly restructure social interaction networks (Singh et al. 2024). Together, these findings suggest that plant-associated resources used by the same insect species in different contexts may shape social interaction networks in distinct ways. Importantly, our null results contribute to a growing recognition that behavioral responses often depend on ecological context and may not always align with theoretical expectations (Popovic et al. 2024). Moreover, recent discussions highlight the importance of publishing such null outcomes to reduce bias and provide more accurate estimates of effect sizes in ecology (Wood 2020; Curry et al. 2025).

Despite limited behavioral responses, dietary treatments strongly influenced survival, with high-carbohydrate diets significantly extending lifespan in both sexes of *A. rosae*. Similar patterns have been observed across a range of insect taxa (Wäckers et al. 2007; Le Couteur et al. 2016), where access to sugar-rich diets prolongs adult survival, including in parasitoid wasps (Cavallini et al. 2023; Lee 2024), butterflies (Molleman et al. 2008) and mosquitoes (Branham et al. 2025). As survival was the only trait profoundly influenced by diet in our study, this highlights that nutritional effects may manifest physiologically even if they are not apparent in behavior. Interestingly, in previous studies on *A. rosae* that were either starved or received honey-water, diet had no significant effect on lifespan (Brueggemann et al. 2025) but had a significant effect on activity (Singh et al. 2023). However, those studies differed from the present one in experimental design, as in the other experiments adults were exposed to a diet of a honey-water mix for up to 4 d only, and honey is compositionally different from the defined nutrient solutions used here.

Across taxa, poor nutritional condition has been associated with riskier behavior, such as higher activity, indicating that nutritional state is a widespread driver of behavioral expression (Moran et al. 2021). In addition, studies of state-behavior feedbacks and consistent individual behavioral variation suggest that energetic or nutritional state can influence not only mean behavior, but also how consistently behavioral differences are expressed among individuals (Sih et al. 2015; Laskowski et al. 2022). In our study, however, diet neither affected activity nor its short-term repeatability, suggesting that such effects may depend on taxon, timescale, or the nutritional axis examined. Nutrition has also been proposed as an important ecological determinant of social interactions, although its effects on sociality can depend on ecological context, including how resources are distributed and how social information is used (Webster and Ward 2011; Lihoreau et al. 2015; Kohles et al. 2022). Thus, the lack of strong dietary effects on the social interaction network structure in our study suggests that short-term differences in single nutrient classes may be sufficient to alter survival, but not necessarily to reorganize levels of interaction under small, standardized group conditions.

Taken together, our results show that variation in the availability and concentrations of single nutrient classes shapes survival but leaves activity and social interaction network structure unmodulated. This divergence suggests that different behaviors, from activity patterns to social interactions (mating and agonistic interactions), to life-history traits, may respond independently to nutritional variation. As adult *A. rosae* forage on nectar of Apiaceae, which have a high natural variation in amino acids and carbohydrates (Venjakob et al. 2022), these findings provide a foundation for linking plant-level nutritional landscapes to insect-level behavioral processes. Future work should examine how variation in complex nutritional environments, such as mixtures of amino acids and carbohydrates or variation in the diversity and composition within each nutrient class, shapes behavioral expression, social interaction networks and survival.

## Supplementary Material

arag102_Supplementary_Data

## Data Availability

Analyses reported in this article can be reproduced using the data provided by Janz et al. (2026).
