# Supplementary material for: Nutrient variation shapes survival without altering activity or social interaction networks in a sawfly
Source: Behav Ecol. 2026 Aug 26;37(6):arag102. doi: 10.1093/beheco/arag102 (PMC13641177; doi:10.1093/beheco/arag102)
Supplement: Supplementary file 1 [file arag102_supplementary_data.docx]

**Supplementary Information**

Table S1. Composition of carbohydrate solutions used in low and high carbohydrate dietary treatments. Values represent the amount of each sugar (in mg) in 50 ml of solution.

| Treatment | Carbohydrate concentration (mg/ml) | Solution volume (ml) | Fructose (mg) | Glucose (mg) | Sucrose (mg) |
| --- | --- | --- | --- | --- | --- |
| Low carbohydrate | 5 | 50 | 125 | 100 | 25 |
| High carbohydrate | 150 | 50 | 3750 | 3000 | 750 |

Table S2. Composition of amino acid solutions used in low and high amino acid dietary treatments. Values represent the amount of each amino acid (in mg) in 50 ml of solution.

| Treatment | Amino acid concentration (mg/ml) | Solution volume (ml) | Alanine (mg) | Arginine (mg) | Aspartate (mg) | Glutamic acid (mg) | Glycine (mg) | Histidine (mg) | Isoleucine (mg) | Leucine (mg) | Lysine (mg) | Serine (mg) | Threonine (mg) | Tyrosine (mg) | Valine (mg) | Methionine (mg) | Phenylalanine (mg) | Proline (mg) |
| --- | --- | --- | --- | --- | --- | --- | --- | --- | --- | --- | --- | --- | --- | --- | --- | --- | --- | --- |
| Low amino acid | 2 | 50 | 3.4 | 3 | 3.2 | 4.4 | 1.4 | 35 | 1.8 | 5.8 | 3 | 7.4 | 1.6 | 1.2 | 2 | 2.8 | 9.6 | 14.6 |
| High Amino acid | 15 | 50 | 25.5 | 22.5 | 24 | 33 | 10.5 | 262.5 | 13.5 | 43.5 | 22.5 | 55.5 | 12 | 9 | 15 | 21 | 72 | 109.5 |

Table S3. Number of adults that reached the social interaction network assay and number that died before the assay, by sex and dietary treatment. Individuals filmed in incomplete groups were counted as having reached the assay, even if they were excluded from the final social interaction network analyses. The proportion of adults dying before the social interaction network assay did not differ significantly among dietary treatments within females (Fisher’s exact test, 𝑃 = 0.870) or males (Fisher’s exact test, 𝑃=0.119).

| Sex | Treatment | Total adults | Reached social interaction network assay | Died before social interaction network assay | Deaths before social interaction network assay % |
| --- | --- | --- | --- | --- | --- |
| Female | Control | 18 | 16 | 2 | 11.1 |
|  | Low amino acid | 16 | 15 | 1 | 6.3 |
|  | High amino acid | 16 | 14 | 2 | 12.5 |
|  | Low carbohydrate | 16 | 15 | 1 | 6.3 |
|  | High carbohydrate | 16 | 16 | 0 | 0 |
| Male | Control | 18 | 13 | 5 | 27.8 |
|  | Low amino acid | 16 | 11 | 5 | 31.3 |
|  | High amino acid | 16 | 14 | 2 | 12.5 |
|  | Low carbohydrate | 16 | 13 | 3 | 18.8 |
|  | High carbohydrate | 16 | 16 | 0 | 0 |

Table S4. Pairwise posterior differences in repeatability (ΔR) among dietary treatments within each sex. Positive values indicate higher repeatability in the first treatment listed in the contrast. Values shown are posterior means with 95% credible intervals and posterior probabilities that ΔR > 0.

| Sex | Contrast | Posterior mean (ΔR) | 95% CrI | Pr(ΔR>0) |
| --- | --- | --- | --- | --- |
| Female | Control − Low amino acid | -0.15 | -0.473 to 0.213 | 0.218 |
| Female | Control − High amino acid | 0.01 | -0.304 to 0.329 | 0.530 |
| Female | Control − Low carbohydrate | 0.05 | -0.324 to 0.428 | 0.606 |
| Female | Control − High carbohydrate | 0.00 | -0.310 to 0.319 | 0.502 |
| Female | Low amino acid − High amino acid | 0.16 | -0.194 to 0.480 | 0.834 |
| Female | Low amino acid − Low carbohydrate | 0.21 | -0.175 to 0.532 | 0.866 |
| Female | Low amino acid − High carbohydrate | 0.15 | -0.198 to 0.456 | 0.813 |
| Female | High amino acid − Low carbohydrate | 0.04 | -0.303 to 0.376 | 0.584 |
| Female | High amino acid − High carbohydrate | -0.01 | -0.300 to 0.283 | 0.466 |
| Female | Low carbohydrate − High carbohydrate | -0.05 | -0.387 to 0.288 | 0.368 |
| Male | Control − Low amino acid | -0.03 | -0.373 to 0.305 | 0.437 |
| Male | Control − High amino acid | -0.02 | -0.364 to 0.328 | 0.465 |
| Male | Control − Low carbohydrate | 0.12 | -0.232 to 0.428 | 0.761 |
| Male | Control − High carbohydrate | 0.00 | -0.326 to 0.331 | 0.508 |
| Male | Low amino acid − High amino acid | 0.01 | -0.368 to 0.380 | 0.533 |
| Male | Low amino acid − Low carbohydrate | 0.15 | -0.202 to 0.486 | 0.804 |
| Male | Low amino acid − High carbohydrate | 0.03 | -0.319 to 0.357 | 0.579 |
| Male | High amino acid − Low carbohydrate | 0.14 | -0.209 to 0.453 | 0.790 |
| Male | High amino acid − High carbohydrate | 0.02 | -0.330 to 0.355 | 0.536 |
| Male | Low carbohydrate − High carbohydrate | -0.12 | -0.430 to 0.210 | 0.224 |

Table S5. Statistical results for (a) mating and (b) agonistic interaction networks. For each response variable, the table reports the test statistic, degrees of freedom, and associated P-value for treatment effects, sex effects, or their interaction. Total numbers of recorded interactions were analysed using negative binomial models; numbers of interacting dyads were analysed using zero-inflated binomial models; and agonistic strength was analysed using a negative binomial mixed-effects model with observation group included as a random effect.

| Interaction | Response variable | Predictor variable | χ² | Df | P-value |
| --- | --- | --- | --- | --- | --- |
| (a) Mating | Number of interactions | Treatment | 3.50 | 4 | 0.477 |
|  | Number of interacting dyads | Treatment | 5.56 | 4 | 0.234 |
| (b) Agonistic | Number of interactions | Treatment | 1.74 | 4 | 0.783 |
|  | Number of interacting dyads | Treatment | 3.17 | 4 | 0.529 |
|  | Strength of interactions | Treatment | 1.71 | 4 | 0.788 |
|  |  | Sex | 1.65 | 1 | 0.199 |
|  |  | Treatment x Sex | 3.26 | 4 | 0.514 |

Table S6. Post-hoc pairwise comparisons among dietary treatments for female lifespan using pairwise contrasts from the Cox proportional hazards model. Values are hazard-ratio estimates (ratio), standard errors (SE), test statistics (z), and Tukey-adjusted p-values. Hazard ratios > 1 indicate higher mortality risk for the first treatment listed in each contrast, and values < 1 indicate lower risk. Significant differences (*P* < 0.05) are shown in bold.

| contrast | ratio | SE | z.ratio | *P* |
| --- | --- | --- | --- | --- |
| Control / Low amino acid | 1.181 | 0.423 | 0.463 | 0.990 |
| Control / High amino acid | 1.184 | 0.408 | 0.489 | 0.988 |
| Control / Low carbohydrate | 1.111 | 0.383 | 0.306 | 0.998 |
| Control / High carbohydrate | 5.639 | 2.25 | 4.328 | **<0.001** |
| Low amino acid / High amino acid | 1.003 | 0.372 | 0.007 | 1 |
| Low amino acid / Low carbohydrate | 0.941 | 0.347 | -0.165 | 0.999 |
| Low amino acid / High carbohydrate | 4.776 | 1.82 | 4.102 | **<0.001** |
| High amino acid / Low carbohydrate | 0.939 | 0.333 | -0.178 | 0.999 |
| High amino acid / High carbohydrate | 4.764 | 1.97 | 3.781 | **0.001** |
| Low carbohydrate / High carbohydrate | 5.075 | 2.07 | 3.977 | **<0.001** |

Table S7. Post-hoc pairwise comparisons among dietary treatments for male lifespan using pairwise contrasts from the Cox proportional hazards model. Values are hazard-ratio estimates (ratio), standard errors (SE), test statistics (z), and Tukey-adjusted p-values. Hazard-ratio interpretation follows the same direction as for females, with ratios > 1 indicating higher mortality risk for the first treatment in the contrast. Significant differences (*P* < 0.05) are shown in bold.

| contrast | ratio | SE | z.ratio | *P* |
| --- | --- | --- | --- | --- |
| Control / Low amino acid | 1.056 | 0.37 | 0.156 | 0.999 |
| Control / High amino acid | 1.124 | 0.386 | 0.34 | 0.997 |
| Control / Low carbohydrate | 1.017 | 0.352 | 0.05 | 1 |
| Control / High carbohydrate | 6.735 | 2.97 | 4.325 | **<0.001** |
| Low amino acid / High amino acid | 1.064 | 0.383 | 0.173 | 0.999 |
| Low amino acid / Low carbohydrate | 0.963 | 0.345 | -0.105 | 1 |
| Low amino acid / High carbohydrate | 6.377 | 2.76 | 4.282 | **<0.001** |
| High amino acid / Low carbohydrate | 0.905 | 0.322 | -0.281 | 0.998 |
| High amino acid / High carbohydrate | 5.992 | 2.68 | 3.996 | **<0.001** |
| Low carbohydrate / High carbohydrate | 6.620 | 2.95 | 4.246 | **<0.001** |

Figure S1. Body mass (mg) of adult *Athalia rosae* across dietary treatments, shown separately for females and males. Boxplots show the median, 25th and 75th percentiles, with whiskers indicating values within 1.5 times the interquartile range. Coloured circles denote individual data points. Body mass differed significantly between the sexes, but not among dietary treatments (ANOVA: sex, $F_{1,158}=157.94$, $P<0.001$; treatment, $F_{4,158}=0.16$, $P=0.959$). Overall, female body mass ranged from 9.8 to 29.7 mg (mean 21.7 mg), whereas male body mass ranged from 4.4 to 23.1 mg (mean 11.9 mg).

Figure S2. Social interaction network for all replicate groups (N = 75), arranged by dietary treatment and plotted with identical layout settings. Each network represents interactions among four adults (two per sex) from the same treatment cohort. Within each treatment panel, replicate networks are arranged in descending order of total interaction frequency. Edge width corresponds to the frequency of interactions within a dyad. Node colours indicate dietary treatment, and node shapes indicate sex. The lighter coloured nodes denote individuals that had no social interactions during our observation period.

Figure S3. Effect of dietary treatment on networks constructed for mating interactions in adult *Athalia rosae*. (A) number of matings and (B) number of mating dyads per group. Boxplots show the median, 25th and 75th percentiles, with whiskers indicating values within 1.5 times the interquartile range. Coloured circles denote individual data points.

Figure S4. Effect of dietary treatment on networks constructed for agonistic interactions in adult *Athalia rosae*. (A) number of agonistic interactions and (B) number of agonistically interacting dyads per group. Individual strength for (C) females and (D) males, respectively. Boxplots show the median, 25th and 75th percentiles, with whiskers indicating values within 1.5 times the interquartile range. Coloured circles denote individual data points.
